# The effect of social environment on bird song: listener-specific expression of a sexual signal

**DOI:** 10.1093/beheco/araa132

**Published:** 2021-03-05

**Authors:** Mónika Jablonszky, Sándor Zsebők, Miklós Laczi, Gergely Nagy, Éva Vaskuti, László Zsolt Garamszegi

**Affiliations:** 1 Institute of Ecology and Botany, Centre for Ecological Research, Alkotmány u. 2–4, 2163 Vácrátót, Hungary; 2 Behavioural Ecology Group, Department of Systematic Zoology and Ecology, ELTE Eötvös Loránd University, Pázmány Péter sétány 1/C, 1117 Budapest, Hungary; 3 The Barn Owl Foundation, Temesvári út 8, 8744 Orosztony, Hungary; 4 MTA-ELTE Theoretical Biology and Evolutionary Ecology Research Group, Institute of Physics, Eötvös Loránd University, Pázmány Péter sétány 1/A, 1117 Budapest, Hungary

**Keywords:** behavioral consistency, mate choice, passerine, territory defense

## Abstract

Animal signals should consistently differ among individuals to convey distinguishable information about the signalers. However, behavioral display signals, such as bird song are also loaded with considerable within-individual variance with mostly unknown function. We hypothesized that the immediate social environment may play a role in mediating such variance component, and investigated in the collared flycatcher (*Ficedula albicollis*) if the identity and quality of listeners could affect song production in signalers. After presenting territorial males with either a female or male social stimulus, we found in the subsequent song recordings that the among-stimulus effects corresponded to non-zero variance components in several acoustic traits indicating that singing males are able to plastically adjust their songs according to stimulus identity. Male and female stimuli elicited different responses as the identity of the female stimuli affected song complexity only, while the identity of male stimuli altered also song length, maximum frequency, and song rate. The stimulus-specific effect on song in some cases decreased with time, being particularly detectable right after the removal of the stimulus and ceasing later, but this pattern varied across the sex of the stimulus and the song traits. We were able to identify factors that can explain the among-stimulus effects (e.g., size and quality of the stimuli) with roles that also varied among song traits. Our results confirm that the variable social environment can raise considerable variation in song performance, highlighting that within-individual plasticity of bird song can play important roles in sexual signaling.

## Teaser

Many animals produce individual-specific signals when communicating with others, but we know relatively little about why such traits also vary within individuals. We demonstrate that males of the collared flycatcher can adjust their songs depending on the identity of their audience, and that several features of song are altered when different male (suggesting opponent-specific investment into territorial aggression) or female (indicating potential male mate choice) stimuli are present in the vicinity.

## INTRODUCTION

Signals in animal communication, especially those under sexual selection, such as bird song, could vary tremendously at multiple levels. The pattern of variation depends also on the type of information conveyed by the signal, for example, high among-individual variability is characteristic to signals of individual identity, but signals of quality are usually moderately variable (Tibbetts et al. 2017). The unique variation of bird song both among and within species (Palmero et al. 2012; Linhart et al. 2013; Favaro et al. 2014) is probably the consequence of its importance, as it can indicate the signaler’s presence, quality, and also identity toward both conspecific males and females (Qvarnström et al. 2010; Vehrencamp et al. 2014; Warrington et al. 2014). Song has an important role in sexual selection, as it primarily functions to deter other males from the owned territory and to attract females as potential mates (Eriksson and Wallin 1986; Lundberg and Alatalo 1992; Catchpole and Slater 2008). To fulfill these signaling functions, song should vary between individuals and be performed with some consistency within individuals to enable the receivers to reliably assess information about the signaler (Boake 1989; Tibbetts and Dale 2007; Schuett et al. 2010). However, song is a behavioral trait, thus it is inherently flexible within individuals in many species and can change according to the environmental and physiological conditions (Gil and Gahr 2002; Arnold et al. 2010), but we do not yet fully know the extent and the causes of this flexibility.

Hence, song traits have dual characteristics, as they include individual-specific components that promote consistency on one hand, and environment-sensitive components that raise within-individual plasticity on the other hand. The relative importance of these components can be statistically described by estimating repeatability (proportion of the phenotypic variance explained by between-individual variance), which appears particularly low for song traits, especially when measured across longer intervals, such as years (Průchová et al. 2017; Zsebők et al. 2017; Naguib et al. 2019). Part of the within-individual variation can be explained by plastic responses to social and non-social environmental effects. Regarding non-social effects, song may be affected, for example, by the inner state of the individual, such as immune state (Garamszegi, Møller, et al. 2004), age (Zipple et al. 2019), reproductive status (Warrington et al. 2014), and breeding experience (Motes-Rodrigo et al. 2017). Songs may also be modified by effects from the external environment, such as territory quality (Hoi-Leitner et al. 1995; Zsebők et al. 2017), predation risk (Schmidt and Belinsky 2013), temperature (Strauß et al. 2020), and it can also depict seasonal variation within an individual (Lattin and Ritchison 2009). As song is used as a signal for conspecifics, social environment may also be an important factor that mediates within-individual variation in song (Geberzahn and Aubin 2014; Gersick and White 2018; Henderson et al. 2018). For example, singers can emit different songs toward males and females in many species (Kroodsma et al. 1989; Kipper et al. 2015; Ronald et al. 2015), but song performance can also depend on the contextual circumstances, such as on the presence of other birds when singing to a female (Vignal et al. 2004; Gersick and White 2018).

Birds may even be able to adjust their songs to the identity or quality of different conspecifics (Heinig et al. 2014) in addition to their sex or status. In fact, the characteristics of the opponent/partner may have both proximate (immediate environmental) and ultimate effects (evolutionary, through indirect genetic effects) on interacting phenotypic traits (such as communication, aggression, or courtship) (Moore et al. 1997; Santostefano et al. 2017). For example, there is evidence for such effects in various taxa in terms of aggression, as the level of aggression of individuals depended on the behavior or on other characteristics of their opponents (Hegyi, Garamszegi, et al. 2008; Wilson et al. 2013; Santostefano et al. 2016). Regarding song, males may benefit from investing more energy, singing more or producing more elaborate songs toward more than less fecund females, if this preferential investment increases the chances of mating with females with higher reproductive potential. Thus, within-individual variation in song quality could reflect such plastic preferences of males as a result of male mate choice evolving when there is a direct benefit of being choosy for the male (Koeninger Ryan and Altmann 2001; Heinig et al. 2014; Fitzpatrick et al. 2018). Furthermore, adjusting song and energy investment to the quality of other males may also be beneficial if the birds can assess the optimal level of investment in territory defense based on the traits of the opponents (Maynard Smith 1982). A well-studied example of this is the phenomenon of “dear enemy” effect, when neighbors with established territory boundaries plastically adjust their songs toward each other to relax unnecessary aggressive encounters between them (Fisher 1954; Temeles 1994; Moser-Purdy and Mennill 2016). However, despite the extent of knowledge on the relationships between bird song and social environment (Glaze and Troyer 2006; Snijders et al. 2015; Gersick and White 2018), few studies have investigated whether the different potential mates or opponents elicit differential response in terms of song production from a particular signaling male (but see Heinig et al. 2014), especially under field conditions.

Our aim here was to investigate the effect of social environment on bird song in a Hungarian population of collared flycatcher (*Ficedula albicollis*), a passerine bird with complex and variable songs that proved to be important traits in sexual selection (Garamszegi, Møller, et al. 2004; Hegyi et al. 2010). We have performed field experiments, in which we systematically varied the contextual background of singing by exposing the focal birds to different social stimuli. In particular, before making song recordings on territorial males, we presented different male or female stimuli on their territory to trigger singing. As we were interested mainly in the effect of stimulus identity, we did not alter the within-stimulus effects and did not use playback during the experiments. If singing males can plastically adjust their songs according to the immediate social environment, we predicted that the identity of the stimulus would explain some variance in some song traits of the singing individual. Given that the above social stimulus is expected to have short-term effects, we also predicted that these effects could be revealed more robustly in songs that are recorded right after the removal of the stimuli than in songs that appear later in the recordings. We also investigated whether some characteristics of the stimulus bird (e.g., size, condition) explains the stimulus-specific effects in the song of the focal male.

## METHODS

### Study site and study species

The study was performed in an oak-dominated forest area in the Pilis-Visegrádi Mountains, Hungary (47°43′N, 19°01′E). The research area belongs to the Duna-Ipoly National Park, and contains about 500 nest boxes, in which the collared flycatcher commonly breeds. Research on birdsong has been carried out since 1999 (Garamszegi, Møller, et al. 2004).

The collared flycatcher is a small, hole-nesting, long-distance migratory passerine. Males arrive earlier in the spring to the breeding grounds and occupy territories that consist of a small area around a nest hole or nest box and they start to sing. There is a sexual dimorphism in plumage, as males are black and white, while females are brownish and white (Cramp and Perrins 1994). Both sexes bear white wing patches of which size is condition-dependent (Török et al. 2003; Hegyi, Rosivall, et al. 2008). Typically, only males have a white forehead patch of which size plays an important role in mate choice (Michl et al. 2002; Hegyi et al. 2010), while the wing patch size is more likely to be used in intrasexual interactions (Garamszegi, Rosivall, et al. 2006; Hegyi, Garamszegi, et al. 2008).

The territorial song performance of the collared flycatcher consists of sequences of songs that are 3–5 s long structures composed of syllables, and are separated from each other by a few second long intervals. The syllable is the smallest unit of the song, which is an around 0.1 s long acoustic feature (Gelter 1987). Collared flycatchers have a moderately high individual repertoire size consisting of 20–100 syllables as could be estimated from 20 songs per individual (Garamszegi, Merino, et al. 2006; Zsebők, Herczeg, et al. 2018). Song traits can serve as individual-specific signals in this species, since these were often found to be correlated with some individual characteristics (Garamszegi et al. 2003; Garamszegi, Merino, et al. 2006; Garamszegi et al. 2007). Song may play important roles in sexual selection in the study species, as it was also associated with estimates of mating success or the degree of male–male competition (Garamszegi, Møller, et al. 2004; Hegyi et al. 2010).

### Field procedures

Data for the present study were collected between 2007 and 2018 during the courtship period of the species, between 11 April and 7 May.

Briefly, we captured male and female birds, presented them to unpaired territorial males and made song recordings after the presentation. We used multiple stimulus birds, as we aimed to test the effect of stimulus identity (as could be assessed from visual cues) on song as follows from our biological hypotheses. Note that for the same reason we did not use playback, as we wanted to avoid inducing further variation within a stimulus.

We first captured male and female birds that were subsequently used to elicit songs from the focal males and to reflect the listener’s perspective (these birds are systemically referred as stimulus birds hereafter), but on plots at least 500 m away from the plots where we made song recordings. Hence, based on the short dispersal distance of this species (Könczey et al. 1992; Jablonszky, Krenhardt, et al. 2020) and the infrequent movement between study plots (Garamszegi, Török, et al. 2004), we can reasonably assume that it was unlikely that the tested males encountered the stimuli previously. However, we cannot entirely exclude this potential confounding effect, but we can argue that the effect of familiarity should cause only some random noise, as we used stimulus birds with more than one focal males. The stimulus birds were captured soon after their arrival, if it was possible (in the case of males), or at most a short time after pairing. Therefore, all birds were captured many days before egg laying, thus they were approximately in the same reproductive stage. The birds used as stimuli were captured and measured in the same way as the tested males (see details below). To avoid any confounding effect arising from male age (e.g., age-dependent plumage characteristics or behavior (Török et al. 2003; Garamszegi, Rosivall, et al. 2006; Evans et al. 2011)), we strictly used only adult males as stimuli. The stimuli were housed in large cages (40 × 24 × 40 cm) with water and food (mealworms) provided ad libitum. The stimulus birds were placed into small cages (15 × 20 × 15 cm) in the morning to use them before making the subsequent song recordings (they were also fed during this period), and then replaced into the housing cages in the end of the day tasks. Altogether, we used 28 female and 20 male stimuli during our study (Table 1). Typically two or maximum three pairs were held in captivity at the same time. We used one stimulus for 1–15 song recordings (mean = 3.33, standard deviation [SD] = 3.02) in total, with 1–5 times in a day (mean = 1.58, SD = 0.88). These variations arose because of the immediate field conditions and logistic constraints. For ethical reasons, we aimed to keep birds in captivity as short as possible, and we could successfully replace them between 1 and 11 days. Before the release of the birds at the site of capture, we verified that they were in prime condition.

**Table 1:** Number of stimulus bird used and sample sizes (number of recordings made) given separately for years and sex of stimulus

	2007	2009	2010	2011	2013	2014	2015	2016	2017	2018
Number of female stimulus used	13	4	3	2	2	1	2	1		
Number of recordings obtained with female stimuli	17	25	4	6	6	1	2	1		
Number of male stimulus used					1	2	4	6	2	6
Number of recordings obtained with male stimuli					1	6	18	29	8	39

In the most active singing period of the day (6:00–12:00) (Pärt 1991; personal observations of the authors), we monitored the study area on a daily basis for newly arrived, unpaired birds and located these displaying males near their occupied nest boxes. Given our standard screening procedures, newly found birds were considered as males having just arrived from the wintering sites. We presented these males with either a male or female bird as social stimulus. The stimuli were used at random, as they became available (because they were also utilized as stimulus in other behavioral tests (Garamszegi et al. 2008)). To mimic the natural situations, females were placed on the top of the nest box (reflecting situation when a mate-sampling female inspects the nest box), while male stimuli were positioned 1.5–2.0 m away from the nest box (mimicking a territorial intrusions) for 5–10 min. The exposition times varied slightly because focal males returned to their territory sooner or later after the disturbance caused by the positioning of the stimulus by the experimenter and due to other constraints on the field. However, we always verified that the focal birds interacted with the stimulus for at least 5 min, assuming that this period was sufficient for the focal male to appropriately perceive the contextual situation. This can also be judged from their behavioral responses, as when returned to their territory focal males immediately started to display their nest-box for female stimuli, or displayed aggressive approaches toward the cage of the male stimuli (Garamszegi et al. 2008). In previous studies, we measured the latency to initiate an aggressive approach toward the male stimulus, but we found that this behavioral variable was weakly, if any, predicted by the identity of the stimulus (Szász et al. 2019). We did not make song recordings during the presentation of the stimuli, as focal males did not typically sing in these situations (they may have uttered some stereotyped contact calls when presented with female stimulus, but these have very little among-individual variance and different function than that of bird song used as a signal in sexual selection). Stimulus birds displayed generally similar behavior throughout all their tests (jumped to and fro in the small cages) and male stimuli never sang during this period.

After the removal of the stimuli, we recorded the song of the focal males using a standard protocol (Garamszegi, Merino, et al. 2006; Garamszegi et al. 2007; Garamszegi et al. 2012; Zsebők, Herczeg, et al. 2018). The sound recordings were made using a Telinga parabola dish with a Sennheiser ME62 microphone and K6 preamplifier on Tascam DR1 and Microtrack II handheld digital recorders (with a 48 kHz sampling rate and 16 bit quality). We only used recordings of unpaired males, for which free-living females were not detected on the territory during the recording. Recordings were only made at relatively good weather conditions without rain and wind, and lasted at least 10 min and included at least 20 songs, to allow the standard estimation of repertoire size (Zsebők et al. 2017). If major disturbance from other birds, such as direct contact with other male or female occurred, the recording was terminated. However, minor, momentarily disturbance from other animals could not be excluded in the field, but it is unlikely that these short-term interruptions decreased considerably the effect of the original stimulus that was presented for several minutes in the immediate vicinity of the nest box of the focal male. However, this potential confounding effect that could not be fully avoided under field conditions should only decrease the effect of the stimulus birds, thus any positive result revealed could be considered robust.

We captured the birds within an hour after the song recordings, by using a spring-trap in their nest boxes for ringing and morphological measurements (our long-term experience and ringing records suggest that recorded birds do not switch territory, so we are highly confident that we captured the bird that had been recorded at the same box). We determined the age of males based on their plumage, since 1-year-old birds bear brown remiges and smaller white patches on their wings, while the remiges of older males are black and their wing patches are larger (Mullarney et al. 1999). The determination of age is not reliable for non-recruit females and as minimum age (which could be determined from the ringing record) could be biased, we avoided the use of this variable in females. Body mass was measured using a Pesola spring balance (with a precision of 0.1 g), tarsus length, size of the wing, and forehead patches were measured with a caliper (with a precision of 0.1 mm). Wing patch size was calculated as the sum of the length of the white area on the outer vanes of the fourth to eighth primaries. Forehead patch size was calculated as the product of the maximum length and width of this white patch (Hegyi et al. 2002; Török et al. 2003). Before the measurements, birds without rings were marked with individually numbered rings (Aranea, Poland) for long-term identification.

All applicable international, national, and/or institutional guidelines for the care and use of animals were followed. Permissions for the fieldwork have been provided by the Middle-Danube-Valley Inspectorate for Environmental Protection, Nature Conservation and Water Management, ref. no’s: KTVF 16360-2/2007, KTVF 30871-1/2008, KTVF 43355-1/2008, KTVF 45116-2/2011, KTVF 21664-3/2011, KTVF 12677-4/2012, KTVF 10949-8/2013, PE/EA/101-8/2018, PE-06/KTF/8550-4/2018, PE-06/KTF/8550-5/2018) and was approved by the ethical committee of the Eötvös Loránd University (ref. no. TTK/2203/3).

### Acoustic analyses

We analyzed song recordings from 60 males after exposure to female stimulus (recorded between 2007 and 2016) and from 84 males after exposure to male stimulus (recorded between 2013 and 2018, Table 1). At the outset of the field seasons from which song recordings originate, we did not intentionally collect repeated measurements with different stimuli, as these recordings were performed independently of the predictions of the current study. Therefore, we do not have repeated data to appropriately estimate the within-individual variance in song due to effects mediated by female stimuli. However, for male stimulus scenarios, we could obtain repeated measurements from 14 focal males (2 recordings from 13 individuals and 3 recordings from 1 individual) from the same breeding season (11 repeats) or from different years (4 repeats), which allowed modeling within-individual effects from the singing males’ perspective.

We characterized each male’s singing performance based on different song traits following the subsequent procedures.

We manually cut out the songs from the recordings using Adobe Audition 3.0 (Adobe Systems) software, choosing 20 good-quality songs for each recording, for which the spectrograms of syllables were clearly distinguishable from the background noise. We used the Ficedula Toolbox (Zsebők, Blázi, et al. 2018) to define the start and endpoints, as well as the minimum and maximum frequencies of each syllable, considering only the dominant frequencies, without the harmonics. These time and frequency boundaries of the defined segments were determined at about 20 dB above the background noise level at spectrographic settings of a Hann FFT window with a 512-point window length and 95% window overlap. From these syllable segments, we extracted five easily measurable spectrographic features automatically with the Ficedula Toolbox: the duration, maximum and minimum frequency, frequency bandwidth and mean frequency of the syllable. The last variable was obtained by taking the peak frequency values in each spectrographic time window and calculating their averages at the syllable level (Garamszegi et al. 2012).

On the level of songs, we measured song length and tempo (the ratio between the number of syllables within song and song length, 1/s). Short-term complexity (hereafter complexity) was calculated as the number of different syllable types/total number of syllables within songs. Additionally, we calculated the minimum, maximum and mean frequency, and the frequency bandwidth of the song based on the mean frequencies of the syllables within the song.

As our focal unit resided at the recording level, we calculated song variables on this hierarchical level using the above song measurements. Accordingly, we averaged all song variables (song length, minimum/maximum/mean frequency, frequency bandwidth, tempo, and complexity) to characterize song at the level of recording. Furthermore, we estimated repertoire size by clustering the syllables into 200 syllable types with k-means method in R with the “kmeans” function in “vegan” R package (Oksanen et al. 2016). To validate the reliability of the clustering method, we compared the estimation of repertoire size of 320 individuals based on manual enumeration (see Zsebők, Blázi, et al. 2018 for the detailed method) with the estimates based on the k-means method. The correlation between the two estimates (Pearson’s correlation, *r* = 0.8, df = 318, *P* < 10^–15^) indicated that the k-means clustering method serves as a reliable surrogate for repertoire size. Therefore, to estimate the repertoire size in our study, we calculated the number of k-mean clusters that could be detected for a given individual based on 20 songs (see also Linossier et al. 2016). We also calculated song rate (the number of songs in a minute calculated as 60/median of song intervals), which inherently corresponded to the same hierarchical level.

For complex traits like bird song, one can define a large number of variables to describe the temporal, structural, and compositional aspects of signal design (Gil and Gahr 2002). The chosen variables defined above correspond to different biological meaning with supposed independence, and are also relevant in sexual selection. Following the practice of our previous studies (Garamszegi et al. 2007, 2008; Zsebők et al. 2017) and to allow comparisons with other species, we deliberately relied on the raw variables instead of combining them in a principal component analysis, which creates artificial products based on statistical constraints that are often hard to interpret biologically. Note that our selection of song variables also involved dimension reduction, in which we relied on biological and not statistical considerations to exclude variables with the same meaning (e.g., the number of syllables in a song may reflect the same information as song length). The statistical independence of the chosen variables can be assessed from their correlation matrix, which is provided in the Supplementary Table S1).

### Statistical analyses

For the majority of the analyses, we used linear mixed models (LMM) to partition the variance components along the predictions of the study. The models were fitted using Maximum Likelihood instead of Restricted Maximum Likelihood method (Bolker et al. 2009). Prior to the analyses and the interpretation of model outputs, the distribution of the variables, as well as model residuals were checked visually by inspecting histograms and q-q plots. Furthermore, homogeneity and homoscedasticity of the residuals, the stability of models against influential data points, as well as absence of collinearity with Variance Inflation Factor (VIF, it was always below 1.12; O’Brien 2007; Freckleton 2011) were also verified before the interpretation of the model outputs. We extracted results from the full models. We calculated 95% confidence intervals (CIs) around the estimates of interest with the help of parametric bootstrapping with 1000 samples (Faraway 2006). Instead of statistical significance, we relied on effect sizes theorem for interpretations (Nakagawa and Cuthill 2007). To obtain effect sizes for the fixed effects in LMM, we conducted likelihood ratio tests (LRT) by comparing the models containing and lacking the respective fixed term of interest, from which we calculated estimates of effect sizes (Cramér’s *V*) based on the χ  ^2^ statistics of the LRT (Cohen 1988) and added the sign of the β estimate to the effect size. We again calculated the 95% CIs with parametric bootstrapping (Nakagawa and Cuthill 2007), and we also added the sign of the β estimate to the effect size here. Since Cramér’s *V* at 1 degree of freedom is equivalent to the correlation coefficient (*r*), effects size of *r* ≈ 0.1 can be considered as small effect, *r* ≈ 0.3 as intermediate effect, and *r* ≈ 0.5 as large effect (Cohen 1988; Møller and Jennions 2002).

To analyze the effect of stimulus identity on song performance, we built series of univariate LMMs using each song trait one by one as response variable in two different analyses corresponding to two independent datasets: one for recordings/traits derived after the presentation of a female and another for recordings/traits derived after a male stimulus. The models included the following explanatory variables as fixed predictors. We controlled for the age of the focal individual, the date of recording and the time elapsed between the removal of the stimulus and start of the song recording (the latter variable can only be entered in the analyses with male stimuli, as for female stimuli such information were not available for most of the cases). We used these fixed effects in these models only to control for their potential effect on the response variables, but we were not interested in their particular explanatory role. The random part of the model included the identity of the stimulus, and we also used year as a random factor, to control for pseudoreplication due to year-specific effects. Given that we used different sets of stimuli in different years, we nested the corresponding random effect term within years. As for some males, we had multiple recordings after stimulating them with different males, we also included the identity of the focal male as an additional random factor in models evaluating the effect of male stimulus.

Those traits, for which we detected that stimulus identity explained non-zero between-individual variance in the above LMMs, were subjected to further analysis. To investigate how the effect of stimuli after their removal changed with time, we created bins of five songs along their original order within a recording and calculated the means of these song traits at the level of these bins (traits were calculated for the 1–5th song, 6–10th song, etc. in the recording). We could not do this analysis for song rate and repertoire size, which were defined at the level of 20 songs.

We were also interested in identifying those stimulus-specific characteristics that cause the among-stimulus variance in song, because we can hypothesize that there are some detectable phenotypic differences among the stimuli that mediate the differential responses from the focal birds. If males sing differently toward different stimuli, this can be because singing males are able to discriminate between the listeners of their song based on some cues that indicate their quality or correlate with the accrued fitness benefit. For example, body size, body condition, or plumage traits can be important traits of the stimuli that can be considered when focal males shape their song performance. Therefore, in cases where we found that the identity of stimulus is associated with non-zero variance in the random part of the LMM, we rerun the same model by adding the characteristics of the stimulus to the list of fixed predictors (see Dingemanse and Araya-Ajoy 2015). Accordingly, we included the following stimulus-specific variables in the model as fixed effects: body size (represented by tarsus length), body condition (residuals from body mass-tarsus regression separately built for the sexes, and we also controlled for year effects by including year as a random factor), wing patch size and forehead patch size (only in males) of the stimuli. We did not use the age of the stimuli even if it may be an important signal of quality, because its determination is unreliable in females, and in the other dataset we strictly used only adult males as stimuli. Time in captivity was also included as an explanatory variable, as it might influence some components of morphology and condition (damage to plumage and gaining weight) as well as the behavior of the stimuli (e.g. less stressed behavior due to habituation to captivity). These continuous predictors were z-transformed because they were on very different scales. The purpose of this modeling exercise was to investigate how the variance explained by the stimuli decreased when the stimulus-specific phenotypic traits were also included in the models as fixed factors. To characterize the magnitude by which the stimulus-specific traits are associated with the song traits, we conducted likelihood ratio tests (LRT) by comparing the models containing/lacking the respective fixed term to calculate effect sizes (Cramér’s *V*) from the χ  ^2^ statistics as described above. Potentially, the behavior or vocalization of the stimuli can also influence the focal birds’ responses, but we did not record these variables during the exposition phase of the experiments. However, the experimental situation was very artificial for the stimuli, and they typically displayed stereotyped behavioral elements with small among-individual variance and no vocalization in the restricted space that were available in the small cage.

All statistical analyses were performed in the R 3.6.1 statistical environment (R Core Team 2019). LMMs were fitted with the “lme4” package (Bates et al. 2011) and model simulations were carried out by the “sim” function from the “arm” package (Gelman and Su 2008). Variance Inflation Factor was calculated by the “vif” function from the “car” package (Fox and Weisberg 2011).

## RESULTS

### Experiments with female stimuli

We found non-zero variance that could be attributed to the identity of the female stimulus only for song complexity (10.94%) when controlling for the considered fixed factors (Table 2). We found a small component of variance to be explained by year effects for song length, maximum frequency and tempo, but overall a large part of the among-recording variance was dumped in the unexplained, residual variance component (89.06–100%, Table 2). For the fixed effects, we report the coefficients in terms of scaled effect sizes and their 95% CIs in Table 2.

**Table 2:** Results from the LMM built for the dataset that corresponds to the experiment using female stimulus

	Fixed effects (signed Cramér’s *V*)		Random effects (variance)		
Song trait	Date of measurement	Age	Female stimulus ID	Year	Residual
Song length (s)	0.158 (−0.089, 0.430)	0.077 (−0.171, 0.359)	0.001 (<0.001, 0.002) 0.26%	0.031 (0.011, 0.098) 8.51%	0.336 (0.248, 0.534) 91.23%
Mean frequency (kHz)	−0.253 (−0.443, 0.007)	−0.395 (−0.472, 0.032)	0 (0, 0)	0 (0, 0)	0.046 (0.034, 0.071) 100%
Minimum frequency (kHz)	−**0.383** (−0.565, −0.143)	−0.095 (−0.367, 0.155)	0 (0, 0)	0 (0, 0)	0.160 (0.121, 0.258) 100%
Maximum frequency (kHz)	−0.015 (−0.299, 0.250)	−0.057 (−0.323, 0.211)	0 (0, 0)	0.008 (0.003, 0.025) 10.66%	0.064 (0.048, 0.100) 89.34%
Frequency range (kHz)	**0.274** (0.080, 0.523)	0.050 (−0.207, 0.318)	0 (0, 0)	0.003 (0.001, 0.008) 1.11%	0.254 (0.189, 0.409) 98.89%
Tempo (1/s)	**0.221** (0.026, 0.510)	0.086 (−0.167, 0.350)	0 (0, 0)	0.008 (0.003, 0.024) 9.08%	0.077 (0.058, 0.122) 90.92%
Complexity	0.069 (−0.199, 0.330)	−0.183 (−0.444, 0.058)	0.0001 (<0.0001, 0.0002) 10.94%	<0.0001 (0, 0)	0.0007 (0.0005, 0.0011) 89.06%
Repertoire size	**0.296** (0.117, 0.542)	**0.287** (0.024, 0.549)	0 (0, 0)	0 (0, 0)	244.300 (182.125, 381.729) 100%
Song rate	0.236 (−0.062, 0.476)	−0.088 (−0.396, 0.218)	0 (0, 0)	0 (0, 0)	2.439 (1.800, 4.209) 100%

Estimates of standardized effect sizes (Cramér’s *V*) with their 95% CIs of fixed effects and variances of the random effects, their 95% CIs and the respective percent of the overall phenotypic variance are given. The sign of the effect size reflects the sign of the β estimate in the model. Effect sizes with CIs excluding 0 are in bold. *N* = 59 (except for song rate, for which *N* = 45).

**Figure 1:**
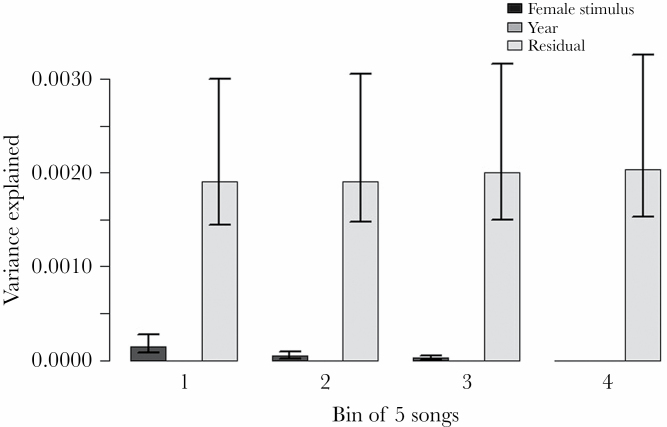
Change in the results of variance partitioning for song complexity after the exposure to female stimuli across different temporal bins of songs (light gray: residual, gray: year, dark gray: female stimulus ID). Bins reflect the temporal arrangement of song recordings each containing five consecutive songs with the first bin having been recorded shortly after the removal of the female. The error bars represent the CIs calculated using parametric bootstrapping

When we analyzed the bins of five consecutive songs for song complexity, we found that the among-stimulus variance decreased along the temporal order of these bins (Figure 1). We provide the statistical outputs of the corresponding models including the variance components and their 95% CIs in the Supplementary Table S2).

When we constructed a LMM for song complexity that also included the phenotypic traits of the female stimuli (tarsus length, body condition, wing patch size, and time in captivity) in the list of fixed predictors, the patterns of variance decomposition in the random part of the model has not changed considerably, and the proportion of variance explained by stimulus identity has not decreased remarkably (10.941% vs. 7.959%). When we investigated the fixed effects, the strength of the relationship between the traits of the stimulus and the song complexity of the focal male covered ranges reflecting small effect sizes (see Supplementary Table S6).

### Experiments with male stimuli

Non-zero variance could be assigned for the identity of male stimuli for song length, maximum frequency, complexity and song rate (8.00–21.47%, Table 3). The identity of the focal male explained 13.69–62.70% of the variance among recordings, except for complexity and repertoire size, where this variance component was estimated to be zero. The effect size estimates for the fixed effects used as control variables together with their 95% CIs are reported in Table 3.

**Table 3:** Results from the LMM built for the dataset that corresponds to the experiment using males as song-stimulus

Song trait	Fixed effects (signed Cramér’s *V*)			Random effects (variance)			
	Date of measurement	Age	Time elapsed until recording	Focal male ID	Male stimulus ID	Year	Residual
Song length (s)	0.210 (−0.006, 0.440)	0.056 (−0.128, 0.269)	−0.042 (−0.261, 0.168)	0.046 (0.032, 0.069) 13.69%	0.031 (0.016, 0.063) 9.16%	0.021 (0.005, 0.094) 6.39%	0.237 (0.188, 0.340) 70.76%
Mean frequency (kHz)	−**0.318** (−0.512, −0.147)	−**0.352** (−0.557, −0.148)	0.008 (−0.216, 0.237)	0.018 (0.015, 0.030) 45.72%	0.001 (<0.001, 0.001) 2.61%	0 (0, 0)	0.021 (0.017, 0.300) 51.67%
Minimum frequency (kHz)	−**0.342** (−0.528, −0.158)	−**0.249** (−0.488, −0.071)	0.088 (−0.133, 0.320)	0.040 (0.030, 0.066) 28.80%	0 (0, 0)	0.0004 (<0.001, 0.001) 0.30%	0.100 (0.079, 0.145) 70.90%
Maximum frequency (kHz)	0.011 (−0.215, 0.235)	0.017 (−0.200, 0.227)	−0.152 (−0.363, 0.062)	0.048 (0.039, 0.070) 52.94%	0.019 (0.010, 0.033) 21.47%	0 (0, 0)	0.023 (0.018, 0.035) 25.59%
Frequency range (kHz)	**0.253** (0.061, 0.458)	0.157 (−0.041, 0.370)	−0.172 (−0.395, 0.036)	0.091 (0.070, 0.148) 34.39%	0 (0, 0)	0.012 (0.002, 0.045) 4.36%	0.162 (0.130, 0.238) 61.26%
Tempo (1/s)	0.033 (−0.181, 0.231)	0.093 (−0.123, 0.316)	−0.050 (−0.289, 0.171)	0.047 (0.042, 0.077) 62.70%	0 (0, 0)	0 (0, 0)	0.028 (0.022, 0.042) 37.30%
Complexity	0.034 (−0.181, 0.259)	−0.190 (−0.412, 0.019)	−0.050 (−0.263, 0.169)	<0.001 (0, 0)	0.0001 (0.0001, 0.0003) 14.47%	0 (0, 0)	0.0008 (0.0006, 0.0012) 85.53%
Repertoire size	**0.218** (0.008, 0.440)	**0.328** (0.114, 0.538)	−0.080 (−0.304, 0.151)	0 (0, 0)	0.005 (0.002, 0.009) 0.002%	37.900 (7.581, 178.412) 14.93%	215.900 (168.373, 307.535) 85.07%
Song rate	**0.228** (0.032, 0.468)	0.047 (−0.178, 0.282)	0.014 (−0.213, 0.258)	1.798 (1.475, 2.768) 50.22%	0.286 (0.110, 0.444) 8.00%	0.005 (<0.001, 0.011) 0.13%	1.492 (1.167, 2.194) 41.66%

Estimates of standardized effect sizes (Cramér’s *V*) with their 95% CIs for fixed effects and variances of the random effects, their 95% CIs and the respective percent of the overall phenotypic variance are displayed. The sign of the effect size reflects the sign of the β estimate of the model. Effect sizes with CIs excluding 0 are in bold. *N* = 87 (except for song rate, for which *N* = 83).

**Figure 2:**
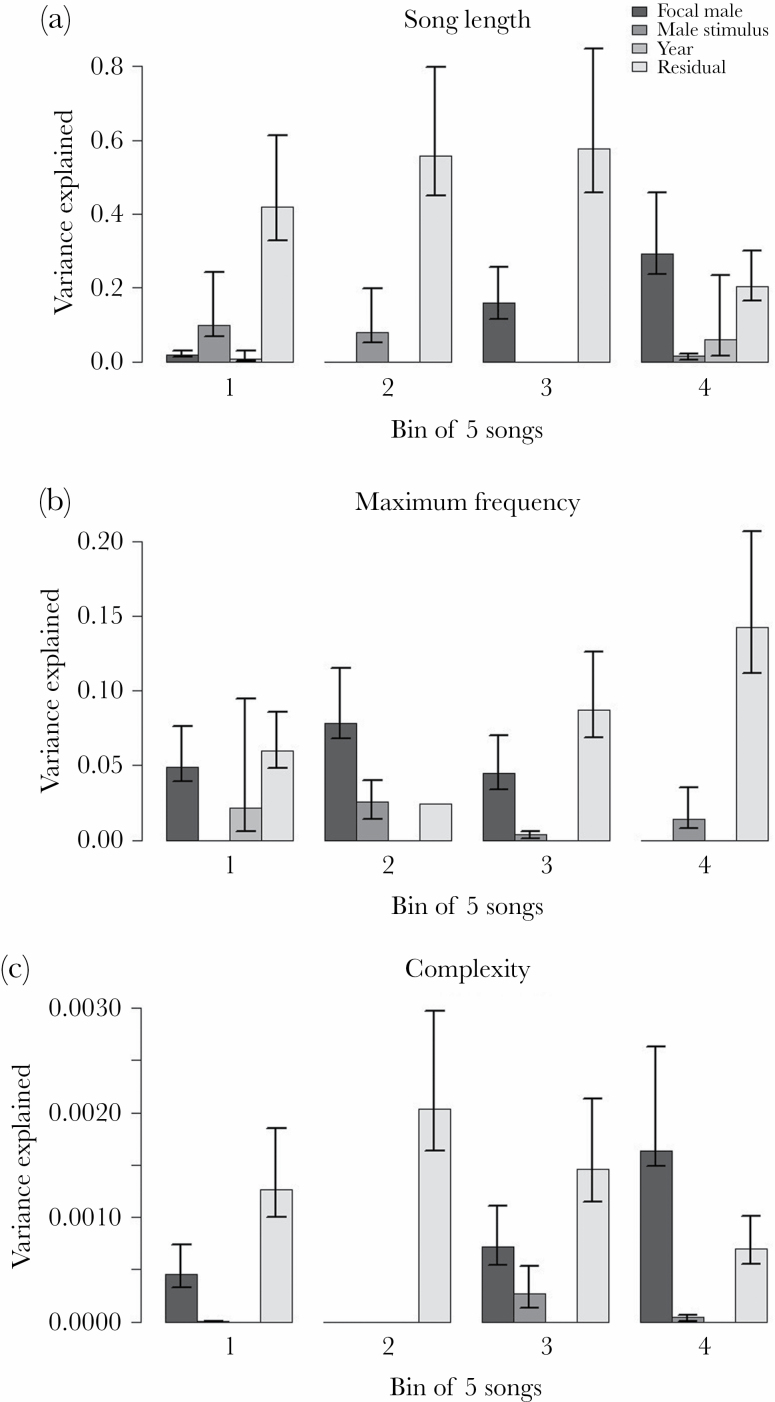
Change in the results of variance partitioning for (a) song length, (b) maximum frequency, and (c) complexity after the exposure of male stimuli across different temporal bins of songs (lightest gray: residual, light gray: year, medium gray: focal male ID, dark gray: male stimulus ID). Bins reflect the temporal arrangement of song recordings each containing five consecutive songs with the first bin having been recorded shortly after the removal of the stimulus. The error bars represent the CIs calculated using parametric bootstrapping

The change in the proportion of variance that is explained by the identity of the male stimulus along temporal windows was less suggestive as it was for female identity in the above model on song complexity. The proportion of explained variance depicted a decreasing tendency along the bins of five songs only for song length, while for the other traits the pattern was more scattered (Figure 2). We provide the statistical outputs of the corresponding models including the variance components and their 95% CIs in the Supplementary Tables S3–S5).

**Figure 3:**
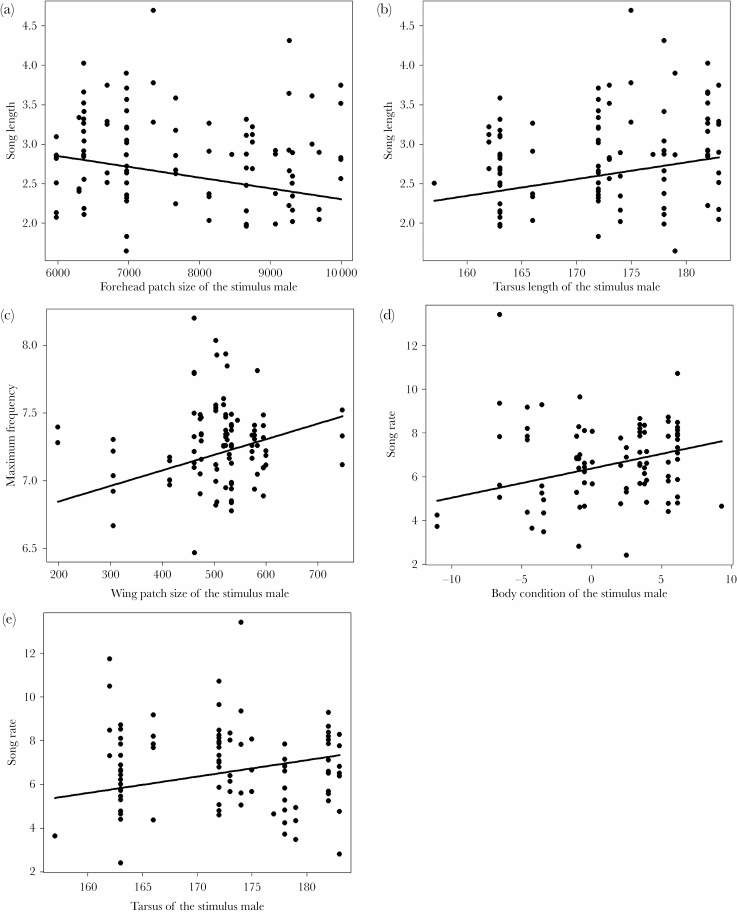
The relationship between the song traits of the focal male and morphological traits of the male stimulus: (a) song length of the focal male and forehead patch size of the stimulus, (b) song length of the focal male and tarsus length of the stimulus, (c) maximum frequency of the focal male and wing patch size of the stimulus, (d) song rate of the focal male and body condition of the stimulus, (e) song rate of the focal male and tarsus length of the stimulus

We investigated further how the characteristics of the male stimuli can mediate the role for the stimulus-specific effects in the random part of the models. We found that the proportion of the among-stimulus variance has been considerably decreased for song length (from 11.232% to <0.001%), maximum frequency (from 22.243% to 4.794%) and song rate (from 12.482% to <0.001%), when the phenotypic traits of the stimulus were included in the model. However, such an influence was less transparent for song complexity (from 16.374% to 10.090%). Overall, the models revealed intermediate effect sizes for i) the negative relationship between the song length of the focal male and forehead patch size of the stimulus (Cramér’s *V* = 0.257, 95% CI = −0.478 to −0.048, Figure 3a); for ii) the positive relationship between the song length of the focal male and the tarsus length of the stimulus (Cramér’s *V* = 0.227, 95% CI = −0.005 to 0.442, Figure 3b); for iii) the positive relationship between the maximum frequency of the focal male and the wing patch size of the stimulus (Cramér’s *V* = 0.236, 95% CI = 0.071–0.531, Figure 3c); for iv) the positive relationship between the song rate of the focal male and the body condition of the stimulus (Cramér’s *V* = 0.269, 95% CI = 0–0.567, Figure 3d); and for v) the positive relationship between the song rate of the focal male and tarsus length of the stimulus (Cramér’s *V* = 0.224, 95% CI = 0–0.528, Figure 3e). The outputs of the corresponding statistical models are given in the Supplementary Tables S7–S10).

## DISCUSSION

The main findings of this study are 4-fold. First, we found that the identity of the stimulus toward which the songs are directed can raise non-zero variance among recordings for several song traits in the collared flycatcher. Second, we detected different patterns (i.e., different roles for different song traits) for the male and female stimuli. Third, the effect of the stimuli in some cases decreased with time, indicating that songs produced shortly after the social stimulus are more affected than songs produced later, but this pattern varied across the sex of the stimulus and particular song traits. Fourth, in the experiments using male stimuli, we were able to identify some of their phenotypic traits that were responsible for the stimulus-specific song responses of the focal male.

Relying on experiments, in which different females were used as the social stimulus, we found that their identity explained non-zero among-recording variance in short-term song complexity of the focal males. Note that in an earlier study of the same population males with lower complexity were paired earlier indicating that the trait could be sexually selected (Hegyi et al. 2010). Although we could only measure some components of the courtship behavior and not the actual preference, the ability of adjusting song to the potential mate would fit with theories on male mate choice suggesting that males would benefit from displaying more elaborately toward females with higher fecundity. Male mate choice has been demonstrated in various taxa like in fishes and arthropods (Pollo et al. 2019; LaPlante and Delaney 2020), but was scarcely investigated and proved in birds (Hill 1993; Wolf et al. 2004; Pryke and Griffith 2007; Holveck et al. 2011). Only a single study focused on song and found that captive Bengalese finch (*Lonchura striata domestica*) males sang with systematically different quantity and quality for different unmated females (Heinig et al. 2014). In this study, we could not identify the female trait that particularly mediates the preferential signaling of males. We could investigate some aspects of female experience, size and condition that could be related to breeding success in terms of laying date and clutch size (Andersson and Gustafsson 1995; Pärt 1995), but we found only weak effect sizes for them. However, we cannot exclude that some unmeasured components of female quality mediate the stimulus-specific effects, which necessitates further investigations. For example age, previous reproductive success, reproductive stage (Ballentine et al. 2003; Naguib et al. 2016), or subsequent parental investment would be obvious candidate traits to study.

When using females as stimulus, their individual effects on complexity were more robust for songs that were recorded immediately after the removal of the stimulus than for songs that were produced later. This temporal pattern is in agreement with the mate sampling behavior of the European black and white flycatchers (Alatalo et al. 1986; Pärt and Gustafsson 1989). In these species, females visit multiple males before pairing to assess the fitness consequences of the underlying breeding opportunity. Hence, the removal of the female stimulus from the males’ territory may simulate the situation when a mate-sampling female leaves the focal male to visit other males. In this context, directed songs toward a particular female may be effective for a relatively short period of time only (see also Heinig et al. 2014).

In the experiments, in which we used males to trigger songs, we found that stimulus-specific effects covered non-zero among-recording variance for several song traits of the focal individual including song length, maximum frequency, complexity, and song rate. The territories are very important resources for flycatcher males playing a major role in female choice (Pärt 1994). Hence, it is not surprising that the presence of a potential competitor on a territory elicits substantial aggressive responses from the territory owner that also includes threat signals. Song as a low-risk aggressive signal may be especially useful in this context (see Garamszegi, Møller, et al. 2004 for the studied population; Linhart et al. 2013; Vehrencamp et al. 2014; Szymkowiak and Kuczynski 2017 for other species). Several examples demonstrate that territory defending males may benefit by adjusting the level of investment in singing to the level of threat the intruder represents (Maynard Smith 1982; Osiejuk and Jakubowska 2017). For instance, red-eyed vireos (*Vireo olivaceus*) uttered more soft songs after playback of stranger songs than after neighbor songs (Moser-Purdy and Mennill 2016). Similarly, playback of calls of an unfamiliar individual triggered stronger call response from cuckoos (*Cuculus canorus*) than the calls of neighbors (Moskát et al. 2017). A list of playback experiments revealed that certain song traits might be altered within individuals in response to the immediate vocal challenge (Benedict et al. 2012; Geberzahn and Aubin 2014; Opaev et al. 2019; Opaev and Kolesnikova 2019). Note that although these playback experiments also targeted within-individual variance in song production, they did not explore the differences in the responses toward different opponents that we achieved here. These findings altogether unanimously emphasize that song traits can be plastically adjusted during a territorial conflict depending on the circumstantial situation, but species-specific roles should be applied regarding the particular traits involved.

We could successfully identify some of those stimulus-specific traits that had elicited different singing response from the territorial males. However, apparently different traits of the stimulus are associated with different song traits indicating that territorial males apply a multidimensional adjustment on their songs based on various aspects of their opponent. In general, the positive and moderately strong relationship between song traits like song length, maximum frequency and song rate of the focal males and the traits of the stimuli like tarsus length, wing patch size, and body condition may imply that collared flycatchers may invest more into songs when faced with an opponent of superior quality. Accordingly, one of these traits, wing patch size of an intruder, was also found to elicit higher level of aggression in the same study population (Garamszegi, Rosivall, et al. 2006). However, an experimentally enlarged forehead patch size increased male–male competition in a Swedish population (Qvarnström 1997), which is in contrast with the expectation based on the negative relationship between song length and the forehead patch size of the stimulus in this study. Other traits with potential impact on song, such as body size, were previously found to play a role in competitive situations in other species (McGhee and Travis 2013; Linhart and Fuchs 2015; Kralj-Fišer et al. 2016).

The roles found in the male–male context were also different from that of the female-male context experiments with regard to the temporal decline of influence. We only found a decreasing tendency in the proportion of variance explained by the identity of male stimuli for song length, but there was no such a clear gradual pattern for maximum frequency and complexity. A possible explanation for this difference between the results of the male- and female-stimulus experiments may be sought in the difference in the underlying contextual situation. During mate sampling, if a female leaves the male’s territory to visit others, quick short-term responses may be more effective. In contrast, during male–male competition, an intruder may be present close to the territory for longer time (especially if it is from a neighboring territory), thus in this context longer sequences of songs might operate as well, leaving less clear temporal patterns. Nonetheless, these results also emphasize the differences in the song responses toward male and female stimuli.

Non-zero variance attributable to the identity of the focal male was found in seven out of the nine acoustic traits, indicating individual-specific song production. In all of these cases, the variance explained by the focal male was greater than the component explained by the male stimulus. These results are comparable to previous repeatability estimates of song in collared flycatchers and other species (Průchová et al. 2017; Zsebők et al. 2017; Naguib et al. 2019).

Overall, we found that different social challenges elicited stimulus-specific responses in various song traits of male collared flycatchers suggesting that these birds (beyond their individual-specific song expression) are also able to flexibly adjust their songs as the immediate social situation requires. Therefore, the interplay between the consistent and plastic variation of bird song has an interesting consequence for the function and evolution of animal signals with different social stimuli differently affecting different song components.

## Supplementary Material

araa132_suppl_Supplementary_Material
