# Supplementary material for: The effect of social environment on bird song: listener-specific expression of a sexual signal
Source: Behav Ecol. 2021 Mar 5;32(3):395–406. doi: 10.1093/beheco/araa132 (PMC8653761; doi:10.1093/beheco/araa132)
Supplement: araa132_suppl_Supplementary_Material [file araa132_suppl_Supplementary_Material.doc]

Supplementary material for The effect of social environment on bird song: stimulus-specific expression of a sexual signal

**Table S1:** correlations between the song variables, the values from the dataset made from recording after the presentation of female stimuli are above the diagonal and the ones after male stimuli are below the diagonal

|  | Song length | Mean frequency | Minimum frequency | Maximum frequency | Frequency range | tempo | complexity | Repertoir size | Song rate |
| --- | --- | --- | --- | --- | --- | --- | --- | --- | --- |
| Song length |  | -0.298 | -0.651 | 0.401 | 0.700 | 0.095 | -0.140 | 0.854 | 0.050 |
| Mean frequency | -0.326 |  | 0.707 | 0.330 | -0.410 | -0.288 | 0.120 | -0.351 | 0.101 |
| Minimum frequency | -0.597 | 0.695 |  | -0.223 | -0.900* | -0.340 | 0.056 | -0.611 | -0.056 |
| Maximum frequency | 0.298 | 0.370 | -0.148 |  | 0.634 | 0.154 | 0.129 | 0.370 | 0.189 |
| Frequency range | 0.615 | -0.318 | -0.836 | 0.667 |  | 0.340 | 0.014 | 0.654 | 0.131 |
| Tempo | -0.076 | 0.226 | 0.111 | 0.195 | 0.025 |  | -0.155 | 0.273 | -0.251 |
| Complexity | -0.320 | 0.090 | 0.066 | -0.105 | -0.108 | -0.439 |  | -0.022 | -0.127 |
| Repertoir size | 0.777 | -0.328 | -0.611 | 0.293 | 0.624 | 0.123 | -0.213 |  | -0.073 |
| Song rate | -0.103 | 0.019 | -0.017 | -0.043 | -0.011 | 0.157 | 0.083 | -0.164 |  |

*An exception from independence is frequency range, which is highly correlated with maximum and minimum frequency, but we argue that these three variables also represent different capacities of the birds

**Table S2**: Variance components with their 95% confidence intervals and the respective percent of the overall phenotypic variance from the models analysing the bins of 5 consecutive songs for song complexity after exposure to female stimuli

|  | Female stimulus ID | Year | Residual |
| --- | --- | --- | --- |
| 1^st^ bin | 0.00014 (0.00008, 0.00028) 7.01% | 0 (0, 0) | 0.00190 (0.00144, 0.00300) 92.99% |
| 2^nd^ bin | 0.00004 (0.00002, 0.00009) 2.26% | 0 (0, 0) | 0.00191 (0.00148, 0.00306) 97.74% |
| 3^rd^ bin | 0.00003 (0.00001, 0.00005) 1.30% | 0 (0, 0) | 0.00200 (0.00150, 0.00316) 98.70% |
| 4^th^ bin | 0 (0, 0) | 0 (0, 0) | 0.00204 (0.00153, 0.00326) 100% |

**Table S3**: Variance components with their 95% confidence intervals and the respective percent of the overall phenotypic variance from the models analysing the bins of 5 consecutive songs for song length after exposure to male stimuli

|  | Focal male ID | Male stimulus ID | Year | Residual |
| --- | --- | --- | --- | --- |
| 1^st^ bin | 0.019 (0.012, 0.028), 3.40% | 0.099 (0.067, 0.241) 18.12% | 0.008 (0.001, 0.028), 1.54% | 0.420 (0.329, 0.613) 76.94% |
| 2^nd^ bin | 0  (0, 0) | 0.079 (0.052, 0.197) 12.40% | 0 (0, 0) | 0.557 (0.449, 0.797) 87.60% |
| 3^rd^ bin | 0.158  (0.114, 0.256),  21.52% | 0 (0, 0) | 0 (0, 0) | 0.577 (0.457, 0.848) 78.48% |
| 4^th^ bin | 0.292 (0.237, 0.457),  50.96% | 0.016 (0.005, 0.021), 2.90% | 0.060 (0.015, 0.235), 10.52% | 0.204 (0.164, 0.301) 35.62% |

**Table S4:** Variance components with their 95% confidence intervals and the respective percent of the overall phenotypic variance from the models analysing the bins of 5 consecutive songs for maximum frequency after exposure to male stimuli

|  | Focal male ID | Male stimulus ID | Year | Residual |
| --- | --- | --- | --- | --- |
| 1^st^ bin | 0.049 (0.039, 0.076), 37.28% | 0 (0, 0) | 0.022 (0.006, 0.095), 16.77% | 0.060 (0.048, 0.086) 45.95% |
| 2^nd^ bin | 0.079  (0.068, 0.115) 61.02% | 0.026 (0.014, 0.040) 20.10% | 0 (0, 0) | 0.024 (0.019, 0.035) 18.88% |
| 3^rd^ bin | 0.045  (0.034, 0.070),  32.88% | 0.004 (0.001, 0.006), 2.76% | 0 (0, 0) | 0.087 (0.069, 0.126) 64.36% |
| 4^th^ bin | 0 (0, 0) | 0.014 (0.008, 0.035), 9.15% | 0 (0, 0) | 0.143 (0.112, 0.207) 90.85% |

**Table S5:** Variance components with their 95% confidence intervals and the respective percent of the overall phenotypic variance from the models analysing the bins of 5 consecutive songs for complexity after exposure to male stimuli

|  | Focal male ID | Male stimulus ID | Year | Residual |
| --- | --- | --- | --- | --- |
| 1^st^ bin | 0.00045 (0.00033, 0.00074) 26.25% | 0.000006 (0.000002, 0.000009) 0.33% | 0 (0, 0) | 0.00126 (0.00100, 0.00185) 73.42% |
| 2^nd^ bin | 0 (0, 0) | 0 (0, 0) | 0 (0, 0) | 0.00204 (0.00164, 0.00297) 100% |
| 3^rd^ bin | 0.00072  (0.00054, 0.00111)  29.40% | 0.00027 (0.00013, 0.00053) 11.10% | 0 (0, 0) | 0.00146 (0.00115, 0.00213) 59.50% |
| 4^th^ bin | 0.00164 (0.00149, 0.00263)  68.69% | 0.00005 (0.00001, 0.00006) 2.02% | 0 (0, 0) | 0.00070 (0.00055, 0.00101) 29.29% |

**Table S6**: Results from the LMM built for complexity in the experiment with female stimulus including the traits of the stimuli. Estimate of β and effect size (Cramér’s V) with their 95% confidence intervals of fixed effects and variance of the random effects and their 95% confidence intervals are displayed. N = 59

| Fixed effects | | | Random effects | |
| --- | --- | --- | --- | --- |
|  | β | Signed Cramér’s V |  | Variance |
| date of measurment | -0.002  (-0.016, 0.012) | -0.041  (-0.322, 0.232) | female stimulus identity:year | 0.00006  (0.00004, 0.00013) |
| age of the focal male | -0.012  (-0.029, 0.007) | -0.176  (-0.454, 0.075) | year | 0  (0,0) |
| time in captivity | <0.001  (-0.011, 0.012) | 0.002  (-0.274, 0.300) | residual | 0.0007  (0.0006, 0.0013) |
| body condition of the stimulus | 0.001  (-0.008, 0.009) | 0.024  (-0.264, 0.304) |  |  |
| tarsus length of the stimulus | -0.003  (-0.012, 0.006) | -0.081  (-0.400, 0.189) |  |  |
| wing patch size of the stimulus | -0.005  (-0.017, 0.006) | -0.127  (-0.404, 0.145) |  |  |

**Table S7:** Results from the LMM built for song length in the experiment with male stimulus, including the traits of the stimuli. Estimate of β and effect size (Cramér’s V) with their 95% confidence intervals of fixed effects and variance of the random effects and their 95% confidence intervals are displayed. N = 82

| Fixed effects | | | Random effects | |
| --- | --- | --- | --- | --- |
|  | β | Signed Cramér’s V |  | Variance |
| date of measurment | 0.178  (0.011, 0.345) | 0.243  (0.022, 0.454) | focal male identity | 0.141  (0.120, 0.249) |
| age of the focal male | 0.129  (-0.172, 0.420) | 0.107  (-0.132, 0.337) | male stimulus identity | <0.001  (<0.001, <0.001) |
| time elapsed until recording | -0.050  (-0.179, 0.089) | -0.087  (-0.342, 0.145) | year | 0  (0, 0) |
| time in captivity | 0.030  (-0.117, 0.186) | 0.046  (-0.179, 0.296) | residual | 0.156  (0.129, 0.245) |
| tarsus length of the stimulus | 0.157  (-0.013, 0.327) | 0.227  (-0.005, 0.442) |  |  |
| body condition of the stimulus | -0.022  (-0.180, 0.128) | -0.034  (-0.262, 0.207) |  |  |
| forehead patch size of the stimulus | -0.175  (-0.332, -0.017) | 0.257  (-0.478, -0.048) |  |  |
| wing patch size of the stimulus | 0.057  (-0.093, 0.200) | 0.090  (-0.136, 0.328) |  |  |

**Table S8:** Results from the LMM built for maximum frequency in the experiment with male stimulus, including the traits of the stimuli. Estimate of β and effect size (Cramér’s V) with their 95% confidence intervals of fixed effects and variance of the random effects and their 95% confidence intervals are displayed. N = 82

| Fixed effects | | | Random effects | |
| --- | --- | --- | --- | --- |
|  | β | Signed Cramér’s V |  | Variance |
| date of measurment | 0.030  (-0.065, 0.134) | 0.068  (-0.221, 0.334) | focal male identity | 0.061  (0.056, 0.103) |
| age of the focal male | 0.055  (-0.084, 0.200) | 0.087  (-0.159, 0.358) | male stimulus identity | 0.004  (0.001, 0.005) |
| time elapsed until recording | -0.048  (-0.106, 0.014) | -0.176  (-0.453, 0.059) | year | 0.006  (0.001, 0.023) |
| time in captivity | -0.004  (-0.083, 0.077) | -0.011  (-0.262, 0.254) | residual | 0.015  (0.013, 0.025) |
| tarsus length of the stimulus | -0.015  (-0.117, 0.085) | -0.032  (-0.296, 0.226) |  |  |
| body condition of the stimulus | 0.017  (-0.068, 0.100) | 0.040  (-0.213, 0.312) |  |  |
| forehead patch size of the stimulus | -0.072  (-0.151, 0.006) | -0.193  (-0.473, 0.020) |  |  |
| wing patch size of the stimulus | 0.103  (0.018, 0.187) | 0.236  (0.071, 0.531) |  |  |

**Table S9: Results from the LMM built for complexity in the experiment with male stimulus, including the traits of the stimuli. Estimate of β and effect size (Cramér’s V) with their 95% confidence intervals of fixed effects and variance of the random effects and their 95% confidence intervals are displayed. N = 82**

| Fixed effects | | | Random effects | |
| --- | --- | --- | --- | --- |
|  | β | Signed Cramér’s V |  | Variance |
| date of measurment | 0.002  (-0.008, 0.013) | 0.035  (-0.182, 0.296) | focal male identity | 0.00007  (0.00005, 0.00012) |
| age of the focal male | -0.014  (-0.028, 0.001) | -0.203  (-0.434, 0.010) | male stimulus identity | 0.00009  (0.00005, 0.00022) |
| time elapsed until recording | -0.001  (-0.008, 0.006) | -0.031  (-0.273, 0.209) | year | 0  (0, 0) |
| time in captivity | -0.002  (-0.011, 0.006) | -0.051  (-0.303, 0.187) | residual | 0.00073  (0.00060, 0.00117) |
| tarsus length of the stimulus | <0.001  (-0.010, 0.010) | 0.003  (-0.242, 0.256) |  |  |
| body condition of the stimulus | 0.002  (-0.007, 0.011) | 0.053  (-0.198, 0.315) |  |  |
| forehead patch size of the stimulus | 0.006  (-0.003, 0.015) | 0.146  (-0.100, 0.413) |  |  |
| wing patch size of the stimulus | -0.005  (-0.014, 0.005) | -0.104  (-0.378, 0.129) |  |  |

**Table S10: Results from the LMM built for song rate in the experiment with male stimulus, including the traits of the stimuli. Estimate of β and effect size (Cramér’s V) with their 95% confidence intervals of fixed effects and variance of the random effects and their 95% confidence intervals are displayed. N = 78**

| Fixed effects | | | Random effects | |
| --- | --- | --- | --- | --- |
|  | β | Signed Cramér’s V |  | Variance |
| date of measurment | 0.730  (0.216, 1.242) | 0.290  (0, 0.594) | focal male identity | 1.720  (1.568, 3.008) |
| age of the focal male | 0.053  (-0.817, 1.019) | 0.014  (-0.222, 0.264) | male stimulus identity | <0.001  (<0.001, <0.001) |
| time elapsed until recording | 0.100  (-0.306, 0.470) | 0.058  (-0.210, 0.361) | year | <0.001  (<0.001, <0.001) |
| time in captivity | -0.348  (-0.769, 0.115) | -0.175  (-0.505, 0.059) | residual | 1.206  (1.015, 2.005) |
| tarsus length of the stimulus | 0.557  (0.098, 1.072) | 0.224  (0, 0.528) |  |  |
| body condition of the stimulus | 0.579  (0.093, 1.053) | 0.269  (0, 0.567) |  |  |
| forehead patch size of the stimulus | 0.271  (-0.216, 0.750) | 0.130  (-0.108, 0.435) |  |  |
| wing patch size of the stimulus | -0.341  (-0.801, 0.131) | -0.165  (-0.480, 0.108) |  |  |
